# Development and Characterization of Nutritious Gluten-Free Doughnuts with Lupin and Inulin Flours

**DOI:** 10.3390/foods11203237

**Published:** 2022-10-17

**Authors:** Hashem AL-Othman, Sofyan Maghaydah, Mahmoud Abughoush, Amin N. Olaimat, Murad A. Al-Holy, Radwan Ajo, Nazieh I. Al Khalaileh, Imranul H. Choudhury, Malak Angor

**Affiliations:** 1 Department of Nutrition and Food Technology, Faculty of Agriculture, Jordan University of Science and Technology, P.O. Box 3030, Irbid 22110, Jordan; hashemalothman87@yahoo.com (H.A.-O.); maghaydah@just.edu.jo (S.M.); 2 Department of Human Nutrition and Dietetics, College of Health Sciences, Abu Dhabi University, Zayed City, Abu Dhabi P.O. Box 59911, United Arab Emirates; 3 Department of Clinical Nutrition and Dietetics, Faculty of Applied Medical Sciences, The Hashemite University, P.O. Box 330127, Zarqa 13133, Jordan; aminolaimat@hu.edu.jo (A.N.O.); murad@hu.edu.jo (M.A.A.-H.); 4 Science of Nutrition and Dietetics Program, College of Pharmacy, Al Ain University, Abu Dhabi P.O. Box 64141, United Arab Emirates; 5 Nutrition and Food Processing Department, Al-Huson University College, Al-Balqa Applied University, As-Salt 19110, Jordan; radwanajo@yahoo.com (R.A.); dr.angormalak@bau.edu.jo (M.A.); 6 Department of Nutrition and Food Science, Faculty of Agriculture, Mu’tah University, Karak 61710, Jordan; nazieh@mutah.edu.jo; 7 College of Pharmacy, Al Ain University, Abu Dhabi P.O. Box 64141, United Arab Emirates; imranul.haq@aau.ac.ae

**Keywords:** celiac disease, doughnuts, gluten-free, inulin, lupin flours

## Abstract

Celiac disease is an immune-mediated disease caused by ingestion of gluten-containing products. The main aim of this study was to develop novel gluten-free doughnuts with high nutritional value using inulin and lupin flour. Five different doughnuts were formulated. Lupin flour was used to replace the potato starch–corn flour composite at levels of 15, 30, 45, 60 and 75% in gluten-free doughnut formulations (AF), (BF), (CF), (DF) and (EF), respectively. Inulin was added to all blends at a level of 6%. Doughnuts made with 100% wheat flour (C1) and 100% corn flour–potato starch blend (C2) served as the controls. The results indicated that the moisture, ash, fat, protein and crude fibre contents of the doughnuts were increased significantly (*p* < 0.05) with increasing levels of lupin flour. The rheological properties showed that the dough development time increased significantly (*p* < 0.05) with increasing lupin flour in the formulation with higher water absorption. The consumer acceptability sensory results varied among the different treatments. However, the AF, CF, and EF doughnuts had the highest value for flavour, texture and crust colour, respectively. Different levels of lupin flour can be used in gluten-free doughnuts production to improve their quality and to enhance their nutritional value in the presence of inulin at a 6% level. These results may have significant implications for the development of novel, healthier food products for gluten-sensitive consumers.

## 1. Introduction

Celiac disease is an immune-mediated disease caused by ingestion of gluten-containing products in genetically susceptible individuals, which results in mucosal inflammation and malabsorption of micro and macronutrients [1]. Gluten is a general term for the storage proteins found in wheat, rye, barley and oats, which are responsible for the desirable physical properties of baked foods, such as elasticity and cohesiveness [2]. Gluten is a complex protein composed of two fractions: gliadins (prolamins) and glutenins. Among these proteins, the gliadins contain similar or repetitive glutamine- and proline-rich peptide sequences that are associated with the toxic aspects of gluten in celiac disease [3]. Celiac disease patients usually experience mucosal destruction, epithelial lymphocytosis and crypt and villous atrophy. Therefore, severe malabsorption of vitamins, minerals, proteins, carbohydrates, and fats may occur [4]. Nutritional deficiencies related to malabsorption in celiac disease can lead to complications, usually more noticeable in children, such as growth abnormalities and short stature. The most effective treatment of celiac disease is a lifelong gluten-free diet [5]. The worldwide market for gluten-free bakery products was valued at USD 5.6 billion in 2020 and it is expected that the market will experience rapid growth and reach USD 7.5 billion in 2027 [6].

Replacement of wheat flour in baked products is a complex challenge because gluten is an essential structure-building protein with important physio-elastic properties. Moreover, gluten-free products are often not fortified and are generally prepared from refined flour and starch. Therefore, the market lacks gluten-free products of high nutritional value. In particular, gluten-free formulas and baked products are generally low in protein; thus, protein fortification of such products is important.

The genus *Lupinus* typically contains 36–52% protein, which is 2–3 times higher than the protein content of cereals. The seeds of white lupin have a protein content ranging from 33% to 47%, depending on genotype and location [7]. Lupin proteins can be classified into water-soluble albumins, salt-soluble globulins, alcohol-soluble prolamins and acid-alkali-soluble glutelins. The main storage proteins of lupin seeds are globulins, which are safe for patients with celiac disease. In contrast to cereals, lupin is a source of sulphur-containing amino acids and arginine and has a good balance of essential amino acids with a high degree of digestibility [8]. The oil content of lupin seeds ranges from 5% to 20%, and the sugar content is ~5.8%. In addition, lupin, like other legumes, is a good source of vitamins and phenolic compounds [9,10].

Inulin is a dietary fibre mainly found in the Asteraceae plant family, as a storage carbohydrate (e.g., Jerusalem artichoke, dahlia and yacon, as well as several edible fruits and vegetables) [11]. The prebiotic properties of inulin are well established. Inulin causes fermentation in the colon, stimulating the growth of health-promoting bacteria such as lactobacilli and bifidobacteria. The main metabolites upon fermentation of inulin are lactate, acetic acid and short-chain fatty acids, such as butyric and propionic acids [12].

To the best of our knowledge, the literature on the inclusion of lupin flour and inulin in gluten-free doughnuts is scarce. Due to the high nutritional properties of lupin, such as its low glycaemic index and high protein, fibre and antioxidant content, and the prebiotic properties of inulin, we hypothesised that enhancing gluten-free doughnuts with lupin flour and inulin would produce a product of high nutritional value for celiac disease patients, with many health benefits. For this reason, the main goal of this study was to improve the nutritional value of gluten-free doughnuts by incorporating inulin and lupin flour. The development of these gluten-free doughnuts of high nutritional value could create a major opportunity for food producers to serve healthier, fibre-rich products for gluten-sensitive consumers.

## 2. Materials and Methods

### 2.1. Materials

Zahra (Patent) wheat flour with an extraction rate of 45%, corn flour, potato starch, sugar and shortening was procured from Modern Flour Mills and Macaroni Factories (Amman, Jordan). White lupin beans (*Lupinus albus*), hydrogenated vegetable shortening, sodium bicarbonate, and salt were purchased from a local market. Inulin was purchased from Guangdong Guanghua Chemical Factory (Shantou, China).

### 2.2. Preparation of Lupin Seeds

The whole grain, white lupin seeds were sorted to remove broken, cracked, or damaged and mouldy seeds. The seeds were then cleaned of dust by immersion in water, then immediately removed and then they were dried for dry milling. The sweet lupin seeds were then milled, using a Labconco mill (Laboratories Construction, Kansas City, MO, USA), into flour until they could pass through a 200 µm screen.

### 2.3. Doughnut Flour Blends and Preparation 

Doughnuts formulated from 100% wheat flour were used as the first control (C1) and the second control (C2) was made from a 100% potato starch–corn flour (1:3 potato starch-to-corn flour ratio) composite. Five different treatments were formulated with lupin flour. Lupin flour was used to replace the potato starch–corn flour composite at a level of 15, 30, 45, 60 and 75% in gluten-free doughnut formulations (AF), (BF), (CF), (DF) and (EF), respectively. The control gluten-free doughnut recipe was established after many preliminary experiments.

The doughnuts were prepared by using sugar (100 g), butter (5 g), sodium bicarbonate (5 g), salt (2 g), one whole egg (60 g), inulin (12 g), and water, as required (according to the farinograph measurements at 500 Brabender Unit (BU) to obtain equal dough consistency). Sugar and butter were mixed in a bowl for 2 min using a mixer at speed four (Kenwood^®^, Havant, UK). The dry ingredients were weighed and mixed with the butter and water at speed three for 3 min to form the doughnut dough. The dough was rolled to a thickness of 1.5 cm and cut using a round steel cutter of 15.0 cm diameter. The cut doughnut dough pieces were deep fried (Stainless Steel Electric Deep Fryer Multifunctional, Germany) using corn oil at 180 °C for 2 min on each side (the oil was changed after frying two batches) and were then allowed to cool at room temperature for 5 min and weighed. All doughnuts were stored in air-tight containers until assessment.

### 2.4. Chemical Analysis

The official methods of analysis of the Association of Official Analytical Chemists [13] were applied for estimating moisture, ash, crude fibre, protein and fat contents. Overall carbohydrates were calculated by difference (i.e., total carbohydrates = 100 − (protein + fat + moisture + ash + crude fibre). The energy content was calculated by multiplying carbohydrate, protein and fat contents by factors of four, four and nine, respectively.

### 2.5. Physical Properties of the Gluten-Free Doughnuts 

#### 2.5.1. Mass, Volume and Density Measurements

The American Association of Cereal Chemists methods were used to evaluate the final core temperature of the doughnuts [14]. The temperature was measured by using a thermocouple (Omega Engineering Inc., model HH21, Stamford, CT, USA), with the probe inserted into the centre of each doughnut immediately after cooking. The probe was inserted into at least three points near the centre of the doughnut to obtain an average temperature measurement. Measurements were performed in triplicate, and the results were expressed as the average value.

The doughnuts were allowed to cool at room temperature on paper towels for ~30 min. Mass and volume were measured immediately after the doughnuts had cooled. Doughnuts were weighed just before frying and at 30 min after frying, to the nearest 0.01 g. The percentage mass change was calculated using Equation (1). The bulk volume of the doughnuts was measured by using the rapeseed displacement method [14] and was performed on both the dough and finished doughnuts to determine the percentage volume change (Equation (2)). The percentage density change was calculated from the mass and volume before and after frying (Equation (3)):

Mass change (%) = ((final mass − initial mass)/initial mass) × 100%(1)

Volume change (%) = ((final volume − initial volume)/initial volume) × 100%(2)

Density change % = ((final density − initial density)/initial density) × 100%(3)

#### 2.5.2. Rheological Characteristics

The water absorption (%) of the composite flour and the dough development time of each blend were evaluated following the AACC54-21 method [14] by using a Brabender farinograph mixer (model 810104, Duisburg, Germany). For the rheological tests, the doughnut dough samples (300 g) were prepared by mixing the composite flour, sugar, salt, inulin and baking powder according to the standard procedure.

#### 2.5.3. Colour Measurement

The colour of doughnut samples was measured by using a Minolta CR-300 colorimeter (Ramsey, NJ, USA) and recorded by using the CIE L*a*b* colour system, where L* is the luminance or lightness component, a* represents green (−a) to red (+a) and b* symbolises blue (−b) to yellow (+b). The colorimeter was calibrated by using a standard white plate. The reference values were L = 97.1, a = +0.13 and b = +1.88. The colour was measured at two positions on both sides of the doughnuts. Triplicate samples of each blend were measured and the average value determined.

### 2.6. Sensory Evaluation

The consumer panel included consumers who often get their doughnuts from the kitchen and graduate students from the Nutrition and Food Technology department at Jordan University of Science and Technology.

Before tasting, panelists signed an informed consent form and they were asked a series of questions about their demographic information (gender, age, and education), frequency of consumption of doughnuts, and food allergies. Individuals were excluded if they reported allergies to any food product.

Their age ranged from 18 to 55 years from both genders, and they came from various socioeconomic backgrounds. A total of 100 participants took part in the trial in order to get the 50 respondents required. Fifty participants were excluded and fifty assessors were retained.

Consumer testing was conducted at the Jordan University of Science and Technology Laboratories. A blind basis method of analysis was applied; the samples codes were done with three-digit numbers. Each consumer was provided with doughnut samples which were presented monadically to panelists on odourless plastic plates at room temperature in a randomised order [15]. Water and unsalted crackers were provided to panelists to cleanse their palates between samples. On a 9-point hedonic scale (1 = dislike extremely to 9 = like extremely) with a neutral point at 5 (neither like nor dislike) consumers were asked to record their: acceptance and intensity scores for overall acceptability, flavour, texture, colour, crust colour, crumb colour, hardness and aftertaste [16].

### 2.7. Statistical Analysis 

The data were analysed using SPSS version 15.0 (Chicago, IL, USA). A one-way analysis of variance (ANOVA) was performed to test for differences between flour blends, followed by separation of the mean values using Duncan’s multiple range tests. Data were considered statistically significant when *p* ≤ 0.05. Data were averages of two repetitions ± standard deviation.

## 3. Results and Discussion

### 3.1. Chemical Analysis of the Flours and Doughnuts

Table 1 presents the chemical composition of the three flour types (wheat, corn and lupin) on a dry weight basis. Lupin flour had significantly higher (*p* ≤ 0.05) levels of crude protein (35.7%), ash (2.88%) and fat (13.1%) than the wheat and corn flours but significantly lower (*p* ≤ 0.05) carbohydrate and moisture levels. These results are in agreement with those obtained by Maghaydah et al. [17], who found that lupin flour had a significantly higher content of protein, ash, fibre, and fat, but the content of carbohydrate was lower compared to wheat, rice, corn and corn starch, which were used to prepare gluten free cookies.

The moisture, ash, fat, protein and crude fibre content of the doughnuts significantly increased (*p* ≤ 0.05) as the amount of lupin flour increased (Table 2). This was directly associated with the chemical composition of lupin flour. The high fibre and protein contents of lupin flour increased the water-holding capacity of the dough, thereby increasing the moisture content of the doughnuts formulated with lupin flour, in concurrence with [18]. Consequently, blend EF, with the highest lupin flour content (75%), contained the highest moisture (20.52%) content. Similarly, the significant (*p* ≤ 0.05) increase in ash content of the doughnuts with increasing lupin flour substitution was attributed to the high fibre (bran) content of lupin flour (Table 2). Bran contains most of the minerals (ash) of the seed. Therefore, increasing the bran content will increase the ash content [19]. Lupin flour also had higher fat (13.10%), protein (35.70%) and crude fibre (9.52%) than corn and wheat flours, as shown in Table 1. Accordingly, blend EF, formulated with the highest level of lupin flour contained the highest fat, protein and crude fibre content. In contrast, the carbohydrate content of the doughnut blends significantly decreased (*p* ≤ 0.05) as lupin flour increased due to its low carbohydrate content (Table 1) [20]. 

Similar results were obtained by the authors of [20], who reported that gluten-free cakes made with lupin flour had more protein, calcium and magnesium than buckwheat cake. Similarly, Hofmanová et al. [21] stated that the use of non-traditional flour composites, such as lupin, can improve the chemical composition of wheat cereal products owing to their high protein, fibre and fat content and other elements beneficial to human health (vitamins, minerals and antioxidants).

### 3.2. Physical Properties of the Gluten-Free Doughnuts

**Figure 1:**
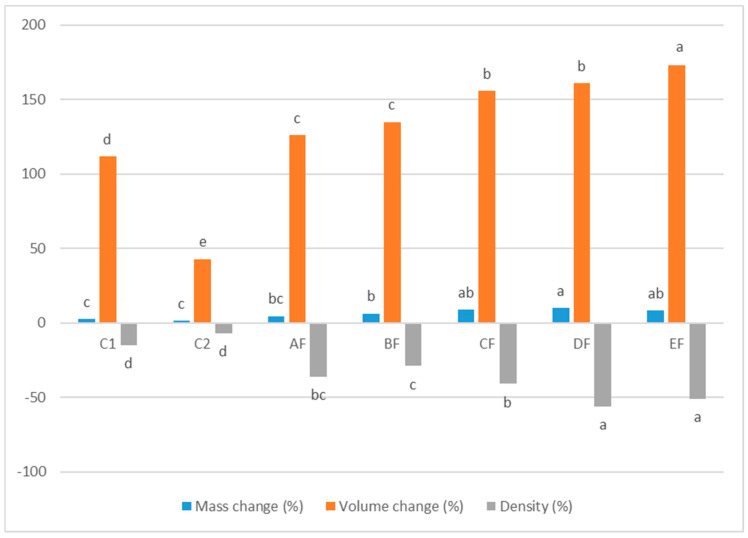
Physical properties of gluten-free doughnut with different treatments: AF (15%), BF (30%), CF (45%), DF (60%) and EF (75%) Lupin flour: potato starch and corn flour composite, (C1) based on wheat flour; (C2) based on potato starch and corn flour composite. Different letters for different treatments mean they are significantly different (*p* ≤ 0.05) based on Duncan’s multiple range test.

The results of the physical property analysis (Figure 1) showed that C2 had significantly lower changes (*p* ≤ 0.05) in mass (1.52%), volume (43%), and density (−7%). CF, DF and EF blends had significantly (*p* ≤ 0.05) higher mass change percentages (8.2–10.3%) than all other blends. EF had the highest volume change (173%). Further, DF and EF had significantly (*p* ≤ 0.05) the highest density change percentages of −56% and −51%, respectively. Higher mass, volume and density changes were observed as the level of lupin flour in the formulation increased. This is because the dough had a higher water content as a direct result of the higher protein content, which increases the water-holding capacity of the lupin flour, which enhances the leavening of bakery products during frying. In addition, incorporation of inulin into lupin-based doughnuts may reduce the amount of absorbed water due to the fact that inulin acted as an oil barrier as well as its high capacity for gas retention. It was found that mass change is associated with the oil uptake of doughnuts [22]. It was reported that the incorporation of carboxymethyl cellulose and xanthan gum as hydrocolloids increased the volume of gluten-free bread based on rice flour, corn starch, and sodium caseinate [23].

### 3.3. Rheological Properties of the Dough

The average water absorption percentage of the blends (Table 3) revealed that as the fraction or level of lupin flour was increased, more water was needed to maintain the dough at 500 BU consistency. An increase in water absorption, following incorporation of lupin flour into wheat flour was reported [24]. The increase in the water absorption capacity of the corn flour-potato starch mix after adding lupin flour is due to its high protein and fibre contents, which compete for water against the other dough constituents [25,26]. Further, inulin Siriwongwilaichat and Kongpanichtrakul [27] found that the moisture content of gluten-free doughnuts increased as the hydrocolloid concentration increased and it was reported that *Jerusalem artichoke* inulin has a water-holding capacity of 4.95 g/g [28].

The dough development time is the time required for the curve to reach its maximum height (i.e., the time needed to reach a consistency of 500 BU). Substituting corn flour–potato starch with lupin flour significantly, (*p* ≤ 0.05) increased the time needed to achieve 500 BU consistency (i.e., longer dough development time) (Table 3) [29]. There was a significant (*p* ≤ 0.05) increasing trend in the dough development time of 8.6, 10.3, 11.4 and 13.6 min at 30, 45, 60 and 75% (BF, CF, DF and EF), respectively, as the potato starch–corn flour combination was substituted with lupin flour. The dough development time was increased with the increase in the level of lupin flour in the blends. This could be attributed to the high protein content. This protein alters the physicochemical properties of the dough, as reported in [29], where the incorporation of lupin protein in wheat flour was studied. Further, it was found that incorporation of up to 10% lupin into wheat flour increased the dough development time [30].

**Figure 2:**
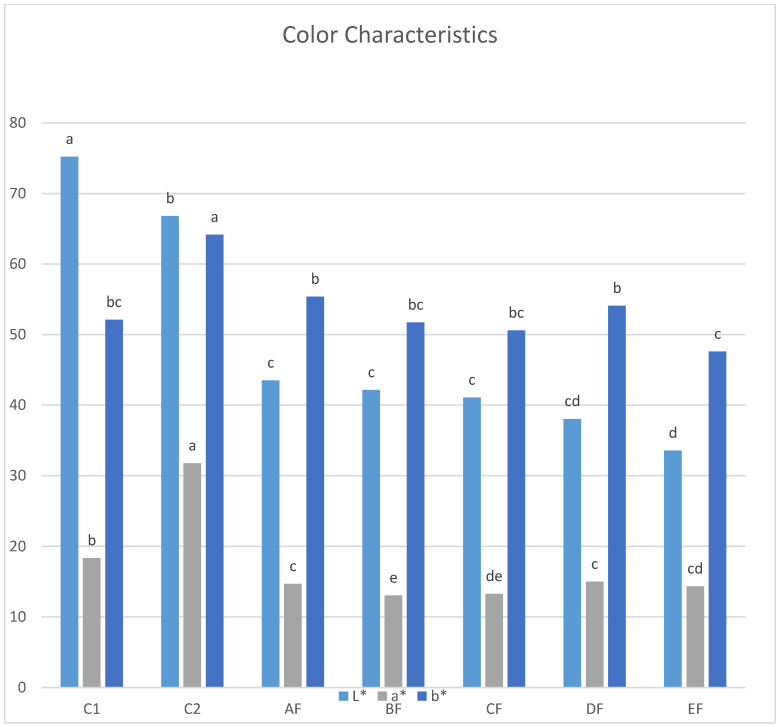
Colour values of gluten-free doughnut with different treatments: AF (15%), BF (30%), CF (45%), DF (60%) and EF (75%) lupin flour: potato starch and corn flour composite, (C1) based on wheat flour; (C2) based on potato starch and corn flour composite. Different letters for different treatments mean they are significantly different (*p* ≤ 0.05) by Duncan’s multiple range test.

Colour Measurement. The L* value of the doughnuts’ crust colour ranged from 33.56 to 75.21 where C1 (100% wheat flour) received the highest L* value, while EF received the lowest L* value (*p* ≤ 0.05). The redness (a*) of the doughnuts’ crust colour ranged from 13.06 for BF to 31.76 for C2 (Figure 2). Yellowness (b*) values of the crust for the doughnut blends (Figure 2) ranged from 47.59 for EF to 64.16 for C2. The difference in the colour characteristics between samples of the blends may be attributed to the differences in the natural pigments in the flours, which in turn depends on the plant [31]. These results were similar to those obtained by Salem and Hanan [32], who found that cakes became darker (lower L*) as the lupin flour quantity increased. In addition, the variations in a and b values among different blends (redness and yellowness) could be due to the variations in their composition (reducing sugar and amino acids), which affect the degree of Maillard browning [33]. Similarly, Mota et al. (2020) found that addition of 10% lupin extract to rice, buckwheat, oat, kamut and spelt flours induced the golden-brown of cookies, and reduced the lightness (L*) value [34].

### 3.4. Sensory Properties of the Gluten-Free Doughnuts

**Figure 3:**
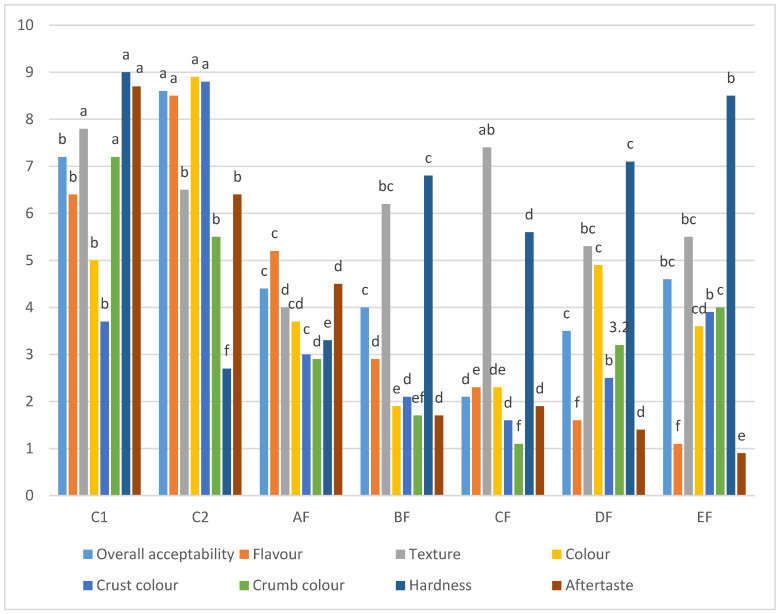
Sensory Attributes of Gluten Free Doughnuts: AF (15%), BF (30%), CF (45%), DF (60%) and EF (75%) lupin flour: potato starch and corn flour composite, (C1) based on wheat flour; (C2) based on potato starch and corn flour composite. Different letters for different treatments mean they are significantly different (*p* ≤ 0.05) based on Duncan’s multiple range test.

The sensory evaluation results are shown in Figure 3. Blend C2 had the highest values for overall acceptability, flavour, colour and crust colour but the lowest hardness values (*p* ≤ 0.05), while blend C1 had the highest values for overall texture, crumb colour, hardness and aftertaste (*p* ≤ 0.05). Blend EF had the lowest scores for flavour and aftertaste (*p* ≤ 0.05). Blend CF had the lowest scores for overall acceptability, crust colour and crumb colour (*p* ≤ 0.05). Blend AF had the lowest score for texture (*p* ≤ 0.05), and blend BF had the lowest score of colour (*p* ≤ 0.05). It was evident that the overall acceptability, flavour, colour, crust colour and aftertaste scores declined as more of the potato starch–corn flour combination was substituted with lupin flour (*p* ≤ 0.05), and this was probably due to increasing levels of alkaloids and proteins in the blend. Alkaloids give a bitter taste, and high protein content leads to undesirable crust and crumb colour due to the Maillard reaction. The overall texture did not significantly (*p* > 0.05) decline as the level of lupin flour was increased. Hardness was significantly (*p* ≤ 0.05) improved as more of the potato starch-corn flour combination was substituted with lupin flour due to more protein in the flour blend. Previous studies on baked products, such as gluten-free cakes and muffins, documented that incorporation of lupin flour at up to 20–30% can be achieved without reducing the sensory quality and acceptability [35]. 

In conclusion, it is possible to produce good quality, high-nutritional-value, gluten-free doughnuts from inulin (6%), potato starch, whole grain corn flour and whole lupin flour at various ratios. However, the gluten-free doughnut formulations yielded lower sensory attributes compared to controls. The doughnut blends with low levels of lupin flour (15–30%) have acceptable flavour and colour. The main challenge in preparing lupin flour-based doughnuts was the bitter taste and unacceptable crust colour. These results have significant implications for the development of healthier, novel gluten-free food products containing lupin flour.

## 4. Conclusions

The results obtained from this study were promising, such that it is feasible to develop quality gluten-free doughnuts with an emphasis on nutritional value regarding increased levels of protein, fiber, and prebiotics and within a sensually acceptable degree by replacing wheat with different levels of lupin flour with the presence of inulin at 6% level. This may have significant implications for the development of a novel, healthier food product for gluten-sensitive consumers.

## Figures and Tables

**Table 1:** Proximate composition of the various flours.

Constituent (%)	Wheat Flour	Corn Flour	Lupin Flour
Moisture	13.4 ± 0.85 ^a^	12.30 ± 0.57 ^a^	8.13 ± 0.52 ^b^
Ash	0.42 ± 0.10 ^c^	1.41 ± 0.23 ^b^	2.88 ± 0.40 ^a^
Fat	1.24 ± 0.18 ^c^	3.08 ± 0.24 ^b^	13.10 ± 1.41 ^a^
Protein ^¥^	12.41 ± 0.44 ^b^	7.86 ± 0.13 ^c^	35.70 ± 2.26 ^a^
Crude fibre	0.13 ± 0.01 ^c^	0.31 ± 0.01 ^b^	9.52 ± 1.36 ^a^
Carbohydrates	72.40 ± 1.72 ^b^	75.04 ± 2.60 ^a^	30.67 ± 2.37 ^c^

¥ Protein (%) was determined by multiplying N (%) by 5.7 for lupin flour and wheat flour and by multiplying N (%) by 6.25 for corn flour. Means in rows with the same superscript letter indicate insignificant differences (*p* ≥ 0.05).

**Table 2:** Proximate composition and energy value of gluten-free doughnuts.

Blends ^§^	Moisture (%) ^‡^	Ash (%)	Fat (%)	Protein (%) ^¥^	Crude Fibre (%)	Carbohydrates (%)	Energy Value (kcal/100 g)
C1	13.22 ± 0.17 ^d^	2.09 ± 0.19 ^c^	8.10 ± 0.30 ^d^	10.21 ± 0.59 ^d^	0.16 ± 0.03 ^f^	66.22 ± 1.60 ^a^	378.62
C2	16.31 ± 0.28 ^c^	2.23 ± 0.06 ^c^	4.90 ± 0.08 ^f^	6.52 ± 0.18 ^f^	1.13 ± 0.06 ^e^	68.91 ± 2.35 ^a^	345.82
AF	16.25 ± 0.23 ^c^	3.30 ± 0.11 ^b^	10.41 ± 0.22 ^c^	14.70 ± 0.51 ^c^	3.72 ± 0.10 ^d^	51.62 ± 1.84 ^b^	358.97
BF	17.20 ± 0.10 ^c^	3.48 ± 0.16 ^b^	10.85 ± 0.13 ^c^	15.31 ± 0.46 ^bc^	4.11 ± 0.04 ^c^	49.05 ± 1.36 ^b^	355.09
CF	18.71 ± 0.44 ^b^	3.55 ± 0.10 ^b^	11.79 ± 0.42 ^b^	15.83 ± 0.61 ^abc^	4.37 ± 0.08 ^b^	45.75 ± 2.11 ^c^	352.43
DF	19.85 ± 0.23 ^a^	3.86 ± 0.08 ^a^	12.04 ± 0.17 ^ab^	16.34 ± 0.38 ^ab^	4.70 ± 0.13 ^a^	43.21 ± 1.06 ^cd^	346.56
EF	20.52 ± 0.81 ^a^	4.15 ± 0.14 ^a^	12.60 ± 0.26 ^a^	17.15 ± 0.92 ^a^	4.81 ± 0.09 ^a^	40.77 ± 0.71 ^d^	345.08

¥ Protein (%) was determined by multiplying N (%) by 6.25 for all treatment samples. ‡ Column values with different superscript letters are significantly different (*p* ≤ 0.05) by Duncan’s multiple range test. § (C1) based on wheat flour; (C2) based on potato starch and corn flour composite; (AF (15%), BF (30%), CF (45%), DF (60%) and EF (75%) “Lupin flour: potato starch and corn flour composite”.

**Table 3:** Farinogram data for the flour blends.

Blends ^§^	Water Absorption (%) ^‡^	Dough Development Time (min)
C1	54.8 ± 1.41 ^e^	3.5 ± 0.42 ^f^
C2	47.3 ± 1.84 ^f^	6.4 ± 0.56 ^e^
AF	66.1 ± 1.56 ^e^	7.1 ± 0.28 ^ed^
BF	73.2 ± 3.82 ^d^	8.6 ± 0.35 ^d^
CF	75.5 ± 1.27 ^cb^	10.3 ± 0.14 ^cb^
DF	80.2 ± 1.13 ^ba^	11.4 ± 0.71 ^b^
EF	81.9 ± 2.12 ^a^	13.6 ± 1.30 ^a^

‡ Column values with different superscript letters are significantly different (*p* ≤ 0.05) by Duncan’s multiple range test. § (C1) based on wheat flour; (C2) based on potato starch and corn flour composite; AF (15%), BF (30%), CF (45%), DF (60%) and EF (75%) lupin flour: potato starch and corn flour composite.

## Data Availability

Data is contained within the article.
