# Correction: AL-Othman et al. Development and Characterization of Nutritious Gluten-Free Doughnuts with Lupin and Inulin Flours. *Foods* 2022, *11*, 3237

**DOI:** 10.3390/foods12091759

**Published:** 2023-04-24

**Authors:** Hashem AL-Othman, Sofyan Maghaydah, Mahmoud Abughoush, Amin N. Olaimat, Murad A. Al-Holy, Radwan Ajo, Nazieh I. Al Khalaileh, Imranul H. Choudhury, Malak Angor

**Affiliations:** 1 Department of Nutrition and Food Technology, Faculty of Agriculture, Jordan University of Science and Technology, P.O. Box 3030, Irbid 22110, Jordan; 2 Department of Human Nutrition and Dietetics, College of Health Sciences, Abu Dhabi University, Zayed City, Abu Dhabi P.O. Box 59911, United Arab Emirates; 3 Department of Clinical Nutrition and Dietetics, Faculty of Applied Medical Sciences, The Hashemite University, P.O. Box 330127, Zarqa 13133, Jordan; 4 Science of Nutrition and Dietetics Program, College of Pharmacy, Al Ain University, Abu Dhabi P.O. Box 64141, United Arab Emirates; 5 Nutrition and Food Processing Department, Al-Huson University College, Al-Balqa Applied University, As-Salt 19110, Jordan; 6 Department of Nutrition and Food Science, Faculty of Agriculture, Mu’tah University, Karak 61710, Jordan; 7 College of Pharmacy, Al Ain University, Abu Dhabi P.O. Box 64141, United Arab Emirates

The authors wish to make corrections to the order of the authors [1].

In the published paper, the order of the author’s names is:

Sofyan Maghaydah ^1,2^, Hashem AL-Othman ^1^, Mahmoud Abughoush ^3,4,^*, Amin N. Olaimat ^3^, Murad A. Al-Holy ^3^, Radwan Ajo ^5^, Nazieh I. Al Khalaileh ^6^, Imranul H. Choudhury ^7^ and Malak Angor ^5^

The correct order of the author’s names should be:

Hashem AL-Othman ^1^, Sofyan Maghaydah ^1,2^, Mahmoud Abughoush ^3,4,^*, Amin N. Olaimat ^3^, Murad A. Al-Holy ^3^, Radwan Ajo ^5^, Nazieh I. Al Khalaileh ^6^, Imranul H. Choudhury ^7^ and Malak Angor ^5^

The authors wish to add the following sentence to the Acknowledgments [1].

**Acknowledgments:** This manuscript was based on the master thesis of Hashem AL-Othman.

The authors apologize for any inconvenience caused and state that the scientific conclusions are unaffected. This correction was approved by the Editor-in-Chief. The original publication has also been updated.
